# Exploring health literacy in patients with chronic kidney disease: a qualitative study

**DOI:** 10.1186/s12882-020-01973-9

**Published:** 2020-07-29

**Authors:** Une Elisabeth Stømer, Astrid Klopstad Wahl, Lasse Gunnar Gøransson, Kristin Hjorthaug Urstad

**Affiliations:** 1 Faculty of Health Science, University of Stavanger, Stavanger, Norway; 2 Department of Nephrology, Stavanger University Hospital, Stavanger, Norway; 3 Faculty of Medicine, University of Oslo, Oslo, Norway; 4 Faculty of Medicine, Department of Clinical Medicine, University of Bergen, Bergen, Norway

**Keywords:** Health literacy, Chronic kidney disease, Patients’ experiences, Qualitative study

## Abstract

**Background:**

Patients with chronic kidney disease make day-to-day decisions about how to self-manage their disease. Chronic kidney disease (CKD) includes a risk for progression towards end-stage renal disease and the development of comorbidities, such as cardiovascular disease, which represents the leading cause of death among these patients. To reduce these risks, CKD patients are recommended to follow a healthy lifestyle with physical activity, food and fluid restrictions, and adherence to complex medication regimes throughout all phases of the disease. To manage the complexity of this health situation, health literacy (HL) is considered essential. The current prevailing understanding is that HL is a multidimensional concept and comprises a range of cognitive, affective, social, and personal skills that determine the motivation and ability to gain access to, understand, and use health information. Recently, we investigated multiple aspects of HL in CKD patients in a quantitative cross-sectional study utilizing the Health Literacy Questionnaire (HLQ) and observed that finding good health information and appraising health information were the most challenging aspects of HL. This study aimed to explore CKD patients’ lived experiences of different dimensions of HL presented in the HLQ.

**Methods:**

This qualitative study utilized in-depth semistructured interviews. Twelve patients with different levels of HL were included. The interviews were analyzed using thematic analysis as described by Braun and Clarke.

**Results:**

We identified three main themes that were significant for CKD patients’ HL: 1. *Variation in people’s attitudes and behavior as health information seekers,* 2. *The problem of fragmented healthcare in the context of multimorbidity makes the healthcare system challenging to navigate*, and 3. *The value of a good relationship with healthcare providers.*

**Conclusion:**

CKD patients take different approaches to health information. Limiting or avoiding health information may be a strategy used by some individuals to cope with the disease and does not necessarily mean that health information is inaccessible or difficult to understand. Comorbidity and a fragmented healthcare system can make the healthcare system challenging to navigate. A good and trusting relationship with healthcare providers seems to promote several aspects of HL and should be promoted to optimize CKD patients’ HL.

## Background

Patients with chronic kidney disease (CKD) make day-to-day decisions about how to self-manage their conditions. Having CKD in the early stages includes a risk for progression towards end-stage renal disease (ESRD) and the development of comorbidities, such as cardiovascular disease, which represents the leading cause of death in this population [1, 2]. Physical symptoms are often diffuse and nonspecific before the disease reaches ESRD [3], which can make CKD easy to neglect in earlier stages. Nevertheless, patients are recommended to follow a healthy lifestyle with physical activity, food and fluid restrictions, and adherence to complex medication regimes throughout all phases of the disease to reduce the risk for progression of kidney disease and the development of comorbidities [3–6]. To manage the complexity of the health situation, adequate health literacy (HL) is considered essential [7, 8]. The current prevailing understanding is that HL is a multidimensional concept and comprises a range of cognitive, affective, social, and personal skills that determine the motivation and ability to gain access to, understand, and use health information [9].

HL in patients with CKD is reported to be limited and to be associated with decreased kidney function and increased hospitalization and mortality rates [10–13]. Low HL is also related to unsound health behavior, such as skipping scheduled dialysis sessions [14], and low immunosuppressant adherence in kidney transplant patients [15]. In addition, patients with lower HL have less access to kidney transplantation [16]. Qualitative research in this field is scarce, but research with other patient groups has indicated that time constraints and medical jargon are common barriers to HL [17–19]. A recent qualitative study in the context of kidney transplantation found that patients needed to be triggered by a symptom or a concern to start seeking health information actively (20). Nevertheless, more research is necessary to understand the concept of HL in the CKD population.

Due to the recent focus on a multifaceted picture of HL, multidimensional assessment tools have been developed [20]. The Health Literacy Questionnaire (HLQ) is one of a few multidimensional assessment tools that cover a range of dimensions, such as personal abilities, social support and accessibilities of the healthcare system [21]. The HLQ has been translated and culturally adapted into more than 30 languages, including Norwegian [22–24], and includes nine domains, which, in turn, facilitate intervention development to improve HL and the assessment of healthcare services [25]. Recent quantitative research utilizing the HLQ in CKD patients revealed that finding and appraising health information seem to be the most challenging dimensions of HL, while cooperating with healthcare providers seems to be least challenging [15, 26, 27]. However, there is discordance between what patients mean and what clinicians mean by their HLQ responses [24], and to better understand patients’ needs, a qualitative approach is necessary.

The aim of this study was, therefore, to gain insight into CKD patients’ experiences of HL based on the domains of the HLQ.

## Methods

### Study design

This qualitative study utilized individual in-depth interviews to gain insight into CKD patients’ experiences with different aspects of HL.

### Setting and context

The current study was part of a broader project aiming to explore HL in CKD patients utilizing both quantitative and qualitative approaches [26]. Participants in the present study had already been included in the first quantitative part of the project. The study participants suffered from CKD stages 3–5 and were receiving treatment at the outpatient clinic or the hemodialysis unit in a Norwegian hospital at the time of the study. The frequency of the appointments varied depending on each participant’s stage and progression of the disease. The outpatient clinic was practicing continuity of care, which means that the patients saw the same nephrologist at each appointment. The hemodialysis unit, on the contrary, did not practice continuity of care due to organizational issues. Hence, dialysis patients saw a variety of nurses and nephrologists during their treatments. The Data Protection Officer at the study hospital approved the study, ID number: 2017/1 [26].

### Data collection and sampling

Twelve patients were invited either by phone or during a scheduled appointment at the hospital to participate in the study. We used maximum variation sampling, which is a purposeful sampling strategy to capture diversity [28–30]. A sample size of twelve participants was considered sufficient to achieve diversity in the level of HL, age, sex, and stage of CKD [30]. Patient characteristics are presented in Table 1. CKD duration was assessed as the time in months from the diagnosis and was recorded upon participants’ inclusion in the study. HL levels were defined based on the participants’ scores on the HLQ from the quantitative part of the project, in which we performed a cluster analysis to identify three different levels of HL: low, medium and high [26]. All of the invited patients agreed to participate in the study and gave written consent before the interviews were conducted.

**Table 1:** Participant characteristics, *n* = 12

Age in years, Median (range)	66 (41–80)
Females, No. (%)	6 (50)
CKD stage, No. (%)	
3	5 (42)
4 and 5, not on dialysis	4 (33)
5, on hemodialysis	3 (25)
Level of HL, No. (%)	
Low HL	5 (42)
Medium HL	3 (25)
High HL	4 (33)
Living alone, No. (%)	5 (42)
Duration of known CKD in months, Median (range)	52 (3–259)
Presence of comorbidity, No. (%)	6 (50)

*CKD* chronic kidney disease, *HL* health literacy

### Data collection/interviews

Data were collected in a private room at the outpatient clinic or the patient’s home from October 2017–March 2018. All the interviews were audiotaped and performed by the same researcher (UES). The interviews lasted from 17 to 48 min (median 35 min) and were transcribed verbatim by the first author and a trained health secretary (MV).

The semistructured interview guide was based on the HLQ, which is based on the World Health Organization (WHO) definition of HL as “the cognitive and social skills which determine the motivation and ability of an individual to gain access to, understand and use information in ways which promote and maintain good health” [31]. The nine aspects of HL included in the HLQ are comprehensively described by Professor Osborne et al. [21]. The interview guide for the current study is presented in Table 2.

**Table 2:** Semistructured interview guide

1	**Opening question** - What do you think of your knowledge of CKD?
2	**About gaining and using health information** - How do you get information about your kidney disease?
3	**About understanding and appraising health information** - What do you do if you cannot understand the information you get? - How do you appraise health information/consider whether health information is relevant to you? - What do you do if you get conflicting recommendations from healthcare providers/others with relevance to your health?
4	**About navigating the healthcare system and managing health** - Can you describe how you navigate the healthcare system? - How do you know when to seek medical assistance? - How do you know who/where to contact? - How do you manage your kidney disease daily (i.e., diet, medications, lifestyle)?
5	**About cooperation and support from healthcare providers** - How do you experience collaboration with healthcare professionals in general? - What do you think about the importance of support from healthcare professionals to manage your disease?
6	**About social support** - Can you explain the role that family and friends have concerning you living with CKD? Others of relevance (peers, others)?
7	**Closing question** - Is there anything we should talk about related to how you are handling CKD that we have not talked about already?

*CKD* chronic kidney disease

### Data analysis

Thematic analysis was used to systematically organize the data into a structured format and to facilitate a deeper understanding of the CKD patients’ experiences of HL. We followed the 6 phases of thematic analysis described by Braun and Clarke [32]. One investigator (UES) generated initial codes from the complete text and further organized the data into meaningful groups (with similar content). The initial codes were not solely related to the nine domains of the HLQ, as data not relevant to HL were also coded. After the initial coding and the organization of the total dataset into meaningful groups, we searched for themes relevant to the domains of the HLQ by the use of mind maps, which resulted in the identification of subthemes and themes. Meaningful groups of data not relevant to HL were not included in the subthemes and themes. An additional investigator (KHU) contributed to organizing the initial codes and extracting them into subthemes and themes. During the analytic process, the four authors (UES, AKW, LGG, and KHU) discussed the themes until we reached consensus. Examples of the analytic process is presented in Table 3.

**Table 3:** Examples of the analytic process, including full citations, initial codes, subthemes, and themes

Citations/raw data	Initial codes	Subthemes	Themes
*118.* *No, but I have chosen not to familiarize myself with it because I don’t want to think about it. Yes, I know that, OK, the nephrologist has me under surveillance; therefore, I do not have to think about it.* *Interviewer: Do you ask about things you wonder about yourself?* *Very seldom, because I know she (the nephrologist) will tell me if there is anything I need to know. I do not read anything.*	Chooses not to familiarize herself with CKD; the nephrologist has her under surveillance, and she does not need to ask questions or think about it.	Passive health information receiver	Variation in people’s attitudes and behavior as health information seekers
*57.* *I got some information from the doctor, and I have read some on the internet myself …*. N*orsk helseinformatikk or something like that or other articles; there is plenty of information out there …*. I*f I find good pages, it is okay, I think …*. *I know my diagnosis, and I look for similar information about it online …*	Obtains some information from the nephrologist and seeks additional information online.	Active health information seeker	
*227.* *I have to say, when you have many different diseases, then it is difficult going to the doctor because they blame it on the other disease; they pass off the responsibility. I think they are frustrated. It is difficult for me to know what the nephrologist’s area is*.	The specialists pass off the responsibility; it is difficult to know which specialist to consult.		The problem of fragmented healthcare in the context of multimorbidity makes the healthcare system challenging to navigate
*118.* *… I feel I have got very good connection with her; I can ask what I want. If there is anything I am concerned about, I ask her; I get answers. If she does not know, she will find out.*	Good connection with the healthcare provider makes it easy to ask about every concern.		The value of a good relationship with healthcare providers

In reporting the data, we follow Braun and Clarke’s 15-point checklist of criteria for good thematic analysis [32].

## Results

The thematic analysis resulted in the identification of three main themes of patients’ experiences relevant to HL: 1. Variation in people’s attitudes and behavior as health information seekers, 2. The problem of fragmented healthcare in the context of multimorbidity makes the healthcare system challenging to navigate, and 3. The value of a good relationship with healthcare providers. The themes revealed both individual and systemic strengths and barriers related to different dimensions of HL.

### **Theme 1.** Variation in people’s attitudes and behavior as health information seekers

The thematic analysis revealed great diversity in participants’ attitudes towards health information seeking. On the one hand, a group of participants described a desire for control and a keen interest in their health conditions. In contrast, other participants expressed a need to protect themselves from the large amount of health information surrounding them, either by actively limiting the information input or by being a passive receiver of health information, waiting for the healthcare provider to give them information. Some were confused about their roles as patients, i.e., whether they were expected to ask questions or simply wait for the healthcare provider to inform them.

Participants characterized as active health information seekers described how they actively utilized available written information material, the internet, and consultations with health care providers to obtain an overview and understanding of their health situations. They expressed that health aspects interested them and that they wanted to learn more. A female participant found it interesting to read about her conditions:

*“I read (about my diseases)! I find it interesting. The more diseases you have, the more interesting it gets” (178. Female (F), 61-70 years, high HL).*

Furthermore, educational sessions held by academic staff were suggested as an excellent opportunity to become engaged in their health situations: “*… I have to be invited to a lecture or something … (The session) shouldn’t have professors or anything like that but should get right down to the care plan ... Make it more attractive for people to get involved in their disease” (99. F, 61–70 years, low HL).*

Internet users seemed to be aware of the risk of junk information and information overload and were selective about which sources of information they accessed. Participants described how they appraised information on the internet and how they were conscious about which websites they accessed in their health information seeking. One of the participants gave an example of one of the resources he relied on:

*“I read Norwegian health informatics or something like that or other articles; there is enough information out there … . If you find good pages, it is okay, I think … . I know my diagnosis, and I look for similar information about it online … ” (57. Male (M), 41-50 years, high HL).*

In contrast to the active health information seekers, some participants seemed to refrain from seeking health information for different reasons. These participants either had a desire to focus on the “*present”* and on “*good things in life”* instead of focusing on the illness and possible long-term problems or trusted the healthcare providers to give sufficient information; therefore, they did not see any reason to search for or read health information. Instead of engaging in information seeking, they expressed that too much focus on health made them feel worse, as described by one male participant:

*“ … I am very conscious about keeping the focus on the present. I have had enough problems associated with diabetes and defibrillators. I am happy to be alive. … . I feel that if I know too much and if I stay in the same environment, it becomes very much like … . pause).* Interviewer: Do you feel sicker? *Yes, I feel like I am getting sicker” (148. M, 41-50 years, low HL).*

Not every participant had a goal of knowing everything about his or her health condition. Two of the participants clearly explained this when describing the amount of health information that was necessary for them:

*“I would just stop and not ask anymore. Because I feel it is enough for me … .” (148. M, 41-50 years, low HL). “ … I am almost 80 years old; it is not that important for me to know everything” (23. M, 71-80 years, low HL).*

These passive information receivers not only refrained from seeking health information but also actively avoided the information. One female participant said she *“moved away” (118. F, 61–70 years, low HL)* when peers started to talk about dialysis treatment at a kidney disease support group because she did not want to hear about it. Another male participant described that he was selective about what he wanted to hear: “… *Some things I want to listen to, and some things I do not want to hear. Yeah, because I do not want to hear about all the bad”(148. M, 41–50 years, low HL).*

The passive information receivers expressed that they relied on healthcare providers to inform them about necessary aspects concerning their health condition. The female participant in the following example admitted having little knowledge about her disease but explained that to avoid continually thinking and worrying, she chose to wait for and trust the healthcare providers to inform her when needed.

*“No, but I have chosen not to familiarize myself with it because I don’t want to think about it. Yes, I know that, OK, the nephrologist has me under surveillance; therefore, I do not have to think about it.****Interviewer*:** Do you ask any questions? *Very seldom, because I know she (the nephrologist) will tell me if there is anything I need to know … I do not read anything” (118. F, 61-70 years, low HL).*

Furthermore, this group of participants showed a skeptical attitude towards seeking health information on the internet for different reasons. First, some expressed being uncertain of their abilities to find appropriate information: “*I am anti-internet or analog” (113. F, 71–80 years, high HL)*; *“No, I am absolutely not searching the internet … there are far too many people searching the internet” (222. M, 61–70 years, medium HL).* Second, the participants described that information on the internet could cause anxiety, which one participant described as follows:

*“I’m not one who reads on the web uncritically. I have quite a few diseases, so I know that starting to read everything online can make you admitted to psychiatric … ” (99. F, 61-70 years, low HL).*

These patients were conscious about protecting themselves from information that could cause anxiety or fear.

Social media, such as Facebook groups where other patients shared their knowledge through lived experiences, was also avoided. A male patient said he had left disease-specific Facebook groups because he had been exposed to ideas from other patients that were harmful for him, for example, the idea that some medications were better than others, and had been encouraged to take medications at doses other than those prescribed by the doctor: “*… It put some ideas in your head, and it is almost spooky … .that’s the reason why I have signed off these pages”(148. M, 41–50 years, low HL).*

Another reason why some patients did not actively seek health information or ask questions was their insecurity about what was expected of them as patients regarding information seeking. A male patient described that his level of knowledge about CKD was low and that he had only short consultations with the nephrologist: “… *Whether it is expected that the patients should ask questions to get information, that I do not know”(148. M, 41–50 years, low HL).*

### **Theme 2.** The problem of fragmented healthcare in the context of multimorbidity makes the healthcare system challenging to navigate

Participants’ experiences of a fragmented healthcare system seemed to make it difficult for them to navigate the healthcare system and to obtain an overview of their health situations. Several participants described having a complex health situation in terms of comorbidities. When being treated in the hospital, some found that the different health specialties and wards had defined responsibilities for their areas only. The fragmented system could result in insecurity regarding which type of health issues should be addressed to which doctor.

*“I have to say, when you have many different diseases, then it is difficult going to the doctor because they blame it on the other disease; they pass of the responsibility. I think they are frustrated. It is difficult for me to know what the nephrologist’s area is” (227. F, 71-80 years, medium HL).*

Sometimes, medical treatment was even contraindicated for another disease, and the participants did not always receive coherent advice:

*“I have psoriasis and psoriasis arthritis. Then, you are told to stay away from beta-blockers. Then, I was in the heart department. They push; they give you beta-blockers. And then you get a conflict; I think it’s wrong. That there is such a poor interconnection” (99. F, 61-70 years, low HL).*

The same participant described how she had learned to “*read between the lines,”* not trusting the healthcare providers to have control of her health situation: “*… I know I have to catch what I can from the different specialists and put it together myself. That’s the way it is …*” *(99. F, 61-70 years, low HL).*

Even though the general practitioner (GP) coordinated the health situation for many patients, some patients preferred consulting nephrologists at the hospital, and a female participant said she was skeptical about consulting the GP after a negative experience of receiving antibiotics that were too strong: “*… Therefore, I have not asked the GP about anything afterward. If I have any concern, I ask the nephrologist” (118. F, 61–70 years, low HL).*

### **Theme 3.** The value of a good relationship with healthcare providers

The participants described how a good and trusting relationship with healthcare providers facilitated better control and understanding of the situation. By seeing the same healthcare provider at each appointment, the participants seemed to feel safe and to develop a low threshold for discussing health issues. The use of informal language made it easy to ask about anything. A good relationship was described in different ways: the healthcare provider and the patient being able to use everyday communication, the patient having a low threshold for asking questions and discussing health issues, the healthcare provider and the patient being at the same level, and the patient having previous positive experiences with the healthcare provider. One patient described that she learned more about her disease after switching to a different nephrologist. She explained that the new nephrologist allowed more time for questions and explained more about the changes in kidney function than the previous nephrologist, resulting in a better understanding of the disease*: “I feel he is teaching much more … he is more open to questions … With simple words … he shows me the development of the renal function and explains … Yes, in fact, I feel like I could be there longer if I wanted …*” *(178. F, 61–70 years, high HL).*

Speaking in informal language and being able to make jokes was appreciated and made it easy to share health concerns:

*“ … to her (the nephrologist), you can say anything … I feel I have got very good connection with her; I can ask what I want. If there is anything I am concerned about, I ask her; I get answers. If she does not know, she will find out” (118. F, 61-70 years, low HL).*

Certain healthcare providers were described as being more natural to talk to in terms of being *“on the same level”* as the patient, and the quality of the relationship was decisive for how much the patients chose to share: “… *but I draw a limit. With some, I share more than with others …*.” *(227. F, 71–80 years, medium HL).*

Patients’ positive former experiences with the healthcare provider, such as the patient being recognized by the nephrologist when admitted to other departments, contributed to the patient developing trust in and a good relationship with the nephrologist. One patient described such a relationship as *“a very safe feeling … he recognized me and took control and adjusted the medications”(148. M, 41-50 years, low HL)*. . Another patient explained, *“She saw me when I was admitted to the emergency room. While I was being examined and taking tests, she suddenly turned up. She was showing interest, and it felt safe …*” *(99. F, 61–70 years, low HL).*

The last quotes illustrate how feeling safe in a vulnerable situation made a significant impression and characterized the CKD patients’ subsequent relationships with healthcare providers.

## Discussion

This study aimed to elicit in-depth insight into CKD patients’ experiences of HL based on the nine domains of the HLQ. Our findings reveal considerable differences in how patients relate to health information and indicate that fragmented healthcare seems to be a barrier to navigating the healthcare system for multimorbid patients. In addition, the relationship with healthcare providers appears to be essential and might compensate for HL challenges.

The diversity in how patients handled health information related to the disease may reflect different coping strategies for patients living with CKD. *Confronting* and *distancing* strategies are two main common strategies for managing a problem, such as coping with chronic disease [33]. Confronting is referred to as a “problem-focused” approach involving taking an active role to resolve or minimize the problem. On the other hand, distancing is an “emotion-focused” approach aimed at regulating emotions such as anxiety and fear attached to the problem [33]. The latter approach is characterized by trying to ignore, trivialize, and not focus on the disease to avoid unpleasant feelings [33]. Previous studies with CKD patients have reported similar findings; for example, an Australian qualitative study characterized CKD patients either as *receivers* (passively seeking) or *engagers* (actively seeking) of health information [34]. In addition, a recent study exploring HL in Norwegian kidney recipients found that the patients fluctuated between different phases in their efforts to balance the amount of information they accessed, suggesting that they needed to be triggered by a symptom or concern to search for health information [35]. Based on our findings and earlier research [34, 35], we suggest that limiting the input of health information might be a strategy for some individuals to cope with CKD. The current study revealed that some patients deliberately *choose* to avoid health information; therefore, providing more information or simplifying existing information might not always be the solution to increase HL.

Another explanation for the variation in how CKD patients relate to health information may relate to the time we live in and the ongoing development of patients’ healthcare services. We are now moving away from a paternalistic healthcare system in which the patients are the passive receivers of healthcare services towards a system in which the patients are supposed to be equal partners with healthcare providers and to contribute to shared decision-making [36–38]. We found that some patients still see the healthcare provider as responsible for their health, viewing themselves as passive receivers of healthcare and health information. Emphasizing the clarification of roles and expectations in the patient-healthcare provider relationship might facilitate better HL in CKD patients.

The presence of comorbidities among CKD patients is high and is associated with adverse outcomes [39, 40]. Compared with coherent care, fragmented care in terms of seeing different healthcare providers with different specialties is associated with higher use of emergency departments, rates of hospitalization, disease progression, and healthcare costs [41–43]. Fragmentation and inconsistency in health recommendations and the challenges of deciding which healthcare provider to contact with different health issues have also been reported to be challenges by earlier qualitative research with patients with CKD [44, 45]. In a recently published study, patients’ suggestions to minimize the consequences of fragmentation included the use of coordinated care, patient education, and self-management support [44]. However, our results show that the desire to be educated varies and that some patients prefer not to know everything about their disease.

We found that a good relationship with healthcare providers characterized by trust and good communication was important for several dimensions of HL, such as sharing health concerns, discussing health issues, and actively engaging with healthcare providers. Earlier studies in various healthcare settings have investigated the importance of the relationship between patients and healthcare providers, revealing that the healthcare provider-patient working alliance is a significant factor in CKD patients’ behavior and a direct predictor of patients’ adherence to treatment and quality of life [46, 47]. Earlier studies have found that indifference and arrogance among healthcare professionals is a barrier to self-care among CKD patients [48]. Based on our findings and previous research, we suggest that ensuring adequate communication skills and facilitating continuity in care with healthcare professionals may promote several aspects of HL in CKD patients.

Our results show that HL in CKD patients is complex and that we need to employ multiple approaches to understand their needs. The qualitative approach in this current study brought nuances to the recent quantitative results from the same population [26], which are important to consider when suggesting clinical implications and further research. The discordance between what patients mean and what healthcare professionals mean by their HLQ responses that was reported in a previous study with other patients [24] also suggests that we need more research to understand the complexity of the concept HL.

### Implications for practice and future research

From a long-term perspective and at the organizational level, creating organizations that are HL responsive (HLR) might facilitate the improvement of HL in CKD patients. An HLR organization refers to an organization that has the flexibility to adapt healthcare services to meet the different needs of the individual patients and the populations they serve [49]. For example, our findings indicate that continuity of care is essential for CKD patients’ HL; hence, an HLR organization can ensure that its outpatient clinic is organized to have sufficient time and staff to provide continuity of care. We recommend that healthcare providers take an individualized approach to each patient since our results indicate that each patient has different needs and preferences that are not always associated with his or her level of HL. More research is necessary to further explore HL in the context of CKD, for instance, the importance of healthcare providers’ communicative skills for CKD patients’ HL and whether the level of HL responds to interventions aimed at improving HL in CKD patients. Earlier research indicate that a conversational tool may be useful in clinical practice to assess strengths and challenges [50]. The quotes in the results section may indicate that patients with low HL are more likely to be passive information receivers, while patients with high HL are more likely to be active health information seekers. It also appears that the level of HL might be associated with the ability to connect with healthcare providers. Due to the aim of the study and the study design, it was not possible to determine whether this association exists; however, it would be interesting to further explore this potential association in future studies. We are aware of an ongoing study with CKD patients at another Norwegian hospital that is investigating the effect of a novel health communication intervention focusing on patients’ roles and capacities as knowledge actors, however the study is not published and there are no preliminary results to report. According to the Clinical Trials web page, there are multiple ongoing studies related to HL and patients with CKD; however, no studies are investigating patient experiences. At a higher level, from a political perspective, a discussion about healthcare systems’ expectations of patients seems to be timely, as the healthcare system is continually evolving, and some patients seem to be confused about their role.

### Strengths and limitations

A strength of this study is the inclusion of patients with different levels of HL based on a quantitative assessment with the HLQ, which ensured diversity in the study sample. There are some limitations to this study. This is a small-scale, single-center study, and hence, the results should be interpreted with caution. More extensive research, including research with more participants from different healthcare institutions and patients with different ethnicities, may have yielded different results. Another limitation is the limited age spread, with no participant under 40 years. However, the current study is highly hypothesis-generating and raises timely questions in an evolving healthcare system.

## Conclusion

CKD patients have different approaches to health information. Limiting or avoiding health information may be a strategy used by some individuals to cope with the disease and does not necessarily mean that health information is inaccessible or difficult to understand. Comorbidity and a fragmented healthcare system can make the health system challenging to navigate. A good and trusting relationship with healthcare providers seems to promote several aspects of HL and should be promoted to optimize CKD patients’ HL.

## Data Availability

The dataset generated and analyzed during the current study is not publicly available due to the individual privacy of the participants but is available from the corresponding author upon reasonable request.

## Abbreviations

CKD: Chronic kidney disease
ESRD: End-stage renal disease
F: Female
GP: General practitioner
HL: Health literacy
HLQ: Health Literacy Questionnaire
M: Male
WHO: World Health Organization
